# Role of zinc substitution in magnetic hyperthermia properties of magnetite nanoparticles: interplay between intrinsic properties and dipolar interactions

**DOI:** 10.1038/s41598-019-54250-7

**Published:** 2019-12-02

**Authors:** Yaser Hadadian, Ana Paula Ramos, Theo Z. Pavan

**Affiliations:** 1 Department of Physics, FFCLRP, University of São Paulo, Av. Bandeirantes, 3900 - Vila Monte Alegre, 14040-900 Ribeirao Preto, SP Brazil; 2 Department of Chemistry, FFCLRP, University of São Paulo, Av. Bandeirantes, 3900 - Vila Monte Alegre, 14040-900 Ribeirao Preto, SP Brazil

**Keywords:** Nanoscience and technology, Nanoscale materials, Physics, Applied physics, Condensed-matter physics

## Abstract

Optimizing the intrinsic properties of magnetic nanoparticles for magnetic hyperthermia is of considerable concern. In addition, the heating efficiency of the nanoparticles can be substantially influenced by dipolar interactions. Since adequate control of the intrinsic properties of magnetic nanoparticles is not straightforward, experimentally studying the complex interplay between these properties and dipolar interactions affecting the specific loss power can be challenging. Substituting zinc in magnetite structure is considered as an elegant approach to tune its properties. Here, we present experimental and numerical simulation results of magnetic hyperthermia studies using a series of zinc-substituted magnetite nanoparticles (Zn_x_Fe_1-x_Fe_2_O_4_, x = 0.0, 0.1, 0.2, 0.3 and 0.4). All experiments were conducted in linear regime and the results were inferred based on the numerical simulations conducted in the framework of the linear response theory. The results showed that depending on the nanoparticles intrinsic properties, interparticle interactions can have different effects on the specific loss power. When dipolar interactions were strong enough to affect the heating efficiency, the parameter *σ* = *K*_*eff*_*V*/*k*_B_*T* (*K*_*eff*_ is the effective anisotropy and *V* the volume of the particles) determined the type of the effect. Finally, the sample x = 0.1 showed a superior performance with a relatively high intrinsic loss power 5.4 nHm^2^kg^−1^.

## Introduction

It was estimated that 9.6 million people worldwide would die from cancer in 2018 and this number is projected to reach over 13 million in 2030^1,2^. Early and accurate diagnosis and effective therapy are key factors for increasing the survival rate. Therefore, in recent decades tremendous efforts were focused on the discovery and development of new diagnostic and therapeutic methods. Magnetic hyperthermia using magnetic nanoparticles has received special attention as a promising therapeutic technique to combat cancer. It aims to selectively raise the temperature of cancerous cells to induce apoptosis and/or necrosis. In addition, hyperthermia can be used to increase the efficiency of conventional therapeutic procedures such as radiotherapy and chemotherapy^3^.

The power dissipated by magnetic nanoparticles exposed to an alternating magnetic field with frequency *f* and amplitude *H*_0_ is converted into heat. This dissipated power (*P*), which is related to the lag between the magnetic response of the material and the external magnetic field, can be expressed as^4,5^:

1 $$P={\int }_{-{H}_{0}}^{+{H}_{0}}{\mu }_{0}M(H)dH,$$

where *µ*_0_ is the permeability of the free space and *M*(*H*) the magnetization of the material. Accordingly, heating efficiency of the magnetic material, typically referred to as specific loss power (SLP), is defined as SLP = *Pf*. Besides the dependence on intrinsic physical properties of the magnetic nanoparticles, SLP is directly proportional to the frequency and amplitude of the magnetic field. However, due to the non-specific heating arising from the induced eddy current in tissues, there is a restriction limit for the frequency and amplitude of applied field. This limit, which is the threshold for major discomfort in the patient and depends on the body area exposed to the magnetic field, was originally^6^ defined as *H*_0_*f* ≤ 4.85 × 10^8^ A·m^−1^ s^−1^. Later, Hergt *et al*.^7^ for smaller radii body parts estimated this limit as *H*_0_*f* ≤ 5 × 10^9^ A·m^−1^·s^−1^. Therefore, optimizing the intrinsic properties of the magnetic nanoparticles to enhance their SLP, respecting this restriction for applied magnetic field, is of considerable importance.

In addition to the applied magnetic field parameters and the intrinsic properties of the magnetic nanoparticles, dipolar interactions can also play an important role in magnetic hyperthermia. Since, in a real colloidal dispersion of magnetic nanoparticles, in addition to the existence of particle aggregation there may always exist a degree of dipolar interactions even in diluted samples^8,9^. This dipolar interactions can sometimes modify the heating efficiency of the system up to two orders of magnitude^8^. Recently, a large number of studies have dealt with the effect of interparticle interactions and apparently a wide range of different theoretical and experimental results have been observed. In most of these papers it is accepted that dipolar interactions are in general “detrimental” for SLP, except when there are particular configurations of the nanoparticles, like chain and similar arrangements in which dipolar interactions can end up increasing the SLP. For example, increase^10–13^, decrease^14–17^, and non-monotonic variation^9,18,19^ in SLP due to the dipolar interaction have been reported. Even constant SLP with sample concentration, which is one of the key factor governing the interparticle interactions, has been observed^20^. In several studies, these results have been interpreted based on morphological parameters such as the shape and size of aggregates, clusters, and chaines formed in the colloids^21,22^, while it has also been shown that the intrinsic magnetic properties of the nanoparticles such as saturation magnetization or anisotropy are key factors in determining how dipolar interaction can affect the heating efficiency^9,18,23^. Therefore, to optimize the heating efficiency of the magnetic nanoparticles a great knowledge of their intrinsic and extrinsic properties is crucial.

Among different magnetic materials considered to be used in biomedical applications including magnetic hyperthermia, spinel ferrite nanoparticles, in particular magnetite and maghemite, are the most popular materials^24,25^. To date, much effort has been devoted to tune their intrinsic properties using different strategies such as modifying the synthesis procedures^24,26,27^ or doping various transition metal cations in their structure^28–30^. Of particular interest is to substitute small amounts of Zn^2+^ for divalent transition metals in the structure of this class of magnetic materials^28,31–35^. Zinc is a diamagnetic cation with zero magnetic moment and has a strong affinity to occupy tetrahedral sites in spinel structure^36^. In magnetite, for example, this weakens the antiferromagnetic coupling between iron cations distributed on both tetrahedral and octahedral sites; therefore, resulting in higher saturation magnetization of the composition. However, by further increase of zinc content, exchange interactions within the octahedral sites would be dominant, leading to spin canting and a decrease in the magnetization of the system^32,37^.

Another interesting feature of doping zinc in ferrite nanoparticles is its effect on nanoparticles’ structure and morphology. It has been shown in several studies that by increase zinc content in ferrites, nanoparticle sizes decrease systematically due to obstructing the crystal growth in spinel structure^38,39^. On the other hand the lattice parameter increases by addition of zinc, mostly due to the site occupancy preference and the difference between the ionic radius of zinc and Fe^3+^ cations in tetrahedral sites^32,39,40^. These changes can, consequently, affect other characteristics of the host nanoparticles such as Curie temperature, blocking temperature, and anisotropy constant^32,41,42^. Therefore, zinc has always been regarded as a promising candidate to tune the magnetic properties of ferrite nanoparticles for biomedical applications, especially for magnetic hyperthermia^25,28,33–35,42–46^.

In our previous study^32^ we reported on the synthesis and characterization of zinc-substituted magnetite nanoparticles (Zn_x_Fe_1-x_Fe_2_O_4_, x = 0.0, 0.1, 0.2, 0.3 and 0.4) as contrast agents in magnetomotive ultrasound imaging. Improving the saturation magnetization, achieved by substitution of zinc in magnetite structure, for this imaging technique played a key role to improve the signal to noise ratio of the images. Addition of zinc in magnetite structure using a simple synthesis method not only modified the saturation magnetization, but also yielded to tailoring other intrinsic properties such as particles’ size and anisotropy; which are all deterministic parameters for magnetic hyperthermia. This can in turn provide a great opportunity to explore the impact of each parameter on the magnetic hyperthermia property of the compositions. In the present paper, we aimed to study the magnetic hyperthermia response of these compositions at a wide range of experimental conditions. Moreover, the effect of dipolar interaction, which strongly depends on intrinsic properties of the particles, was investigated by changing the concentrations of the samples. Finally, the SLP values in the framework of the linear response theory were numerically simulated to infer the effect of dipolar interactions results.

## Methods

### Theory

Successful analytical description of the power dissipation, considering a dispersion of non-interacting nanoparticles with randomly oriented magnetic moments, has been developed in linear regime wherein the condition *µ*_0_*H*_0_*M*_*s*_*V* ≤ *k*_*B*_*T* is satisfied and the magnetization is linearly related to the applied magnetic field (*M* = *χH*)^5,47,48^. Here, *M*_*s*_ is the saturation magnetization, *V* the volume and $\chi =\chi ^{\prime} -i\chi ^{\prime\prime}$ the complex susceptibility of the nanoparticles, *k*_*B*_ the Boltzmann constant, and *T* the absolute temperature. Indeed, $\chi ^{\prime}$ is the in-phase and $\chi ^{\prime\prime}$ the out-of-phase component of the susceptibility also known as the loss component. In this framework, dissipated power is described as:

2 $$P=\pi {\mu }_{0}{H}_{0}^{2}f\chi ^{\prime\prime} \,\,{\rm{where}}\,\chi ^{\prime\prime} =\frac{2\pi f\tau }{1+{(2\pi f\tau )}^{2}}{\chi }_{0},$$

In Eq. (2), ${\chi }_{0}$ is the static susceptibility and can be defined as ${\mu }_{0}{M}_{s}^{2}V/3{k}_{B}T$, *τ* is the effective relaxation time given by $\tau ={\tau }_{N}{\tau }_{B}/({\tau }_{N}+{\tau }_{B})$, in which *τ*_*N*_ and *τ*_*B*_ are the Néel and Brownian relaxation times, respectively, and defined as^5^:

3 $${\tau }_{N}={\tau }_{0}\exp ({E}_{A}/{k}_{B}T),$$

4 $${\tau }_{B}=3\eta {V}_{h}/{k}_{B}T,$$

where *τ*_0_ is the inverse attempt frequency which depends on a variety of intrinsic properties such as saturation magnetization and magnetic anisotropy and can have values between 10^−13^–10^−8^ s^14,49^. *E*_*A*_ = *K*_*eff*_*V* is the energy required to flip the magnetic moment of the particle or the activation energy barrier which separates two opposite directions of magnetic moment in a single domain particle with an uniaxial anisotropy. Here, *K*_*eff*_ is the effective anisotropy which can have different contributions from sources such as magnetocrystalline and surface anisotropy. In Eq. (4), *η* is the viscosity of the medium and *V*_*h*_ the hydrodynamic volume of the nanoparticles. Finally, SLP can be calculated using Eq. 2 as SLP = *P/ρ*, where *ρ* is the density of the magnetic nanoparticles. Numerical simulations of SLP in the framework of the linear response theory were conducted to aid interpretation of experimental data. Variation of the SLP values with respect to the dimensionless parameter *σ* = *K*_*eff*_*V /k*_*B*_*T* was investigated to understand the heating efficiency of the samples.

Moreover, the dynamic hysteresis loop of the magnetic nanoparticles in an oscillating magnetic field $H(t)={H}_{0}\,\cos (2\pi f)$ was also calculated by^5^:

5 $$M(t)=\frac{{\chi }_{0}}{{(1+{(2\pi f\tau )}^{2})}^{1/2}}{H}_{0}\,\cos (2\pi ft+\varphi ),$$

where $\varphi =\arctan (2\pi f\tau )$ is the phase lag between the magnetization and the external field.

### Magnetic nanoparticles

Zinc-substituted magnetite nanoparticles (Zn_x_Fe_1-x_Fe_2_O_4_, x = 0.0, 0.1, 0.2, 0.3 and 0.4) were prepared using the coprecipitation method. Their sizes were determined by transmission electron microscopy, TEM, (JEOL JEM- 100 CXII instrument) and vibrating sample magnetometer (QUANTUM DESIGN MPMS® SQUID VSM DC magnetometer) was used to characterize their magnetic properties. Details about synthesis and characterization of the magnetic nanoparticles are fully presented in^32^. The hydrodynamic size and the zeta-potential of the particles were measured by dynamic light scattering (DLS) and using a Zeta-Sizer system (Malvern Instruments). The Brownian relaxation times were calculated using the obtained sizes from DLS measurements and Eq. (4). In order to estimate the effective anisotropy constant of the samples, at sufficiently high magnetic fields, we fitted the magnetization curves using the law of approach to saturation^50^ as:

6 $$M={M}_{s}(1-\frac{a}{H}-\frac{b}{{H}^{2}})+cH,$$

where $b=\beta {K}_{eff}^{2}/{\mu }_{0}^{2}{M}_{s}^{2}$ and for magnetic nanoparticles with uniaxial anisotropy, *β* is 4/15^51^. Then, the Néel relaxation time of each sample was calculated using Eq. (3).

### Magnetic hyperthermia experiments

The magnetic field was generated using a homemade frequency adjustable magnetic hyperthermia system. A solenoid coil (14 mm in diameter and 87 mm in height) capable of generating a homogenous field in the volume of 6.8 cm^3^, ensuring the whole sample is immersed in a region with uniform amplitude, was used. Details about design, construction, and characterization of the system has been published elsewhere^52^.

For the calorimetric measurements of SLPs, we, initially, prepared aqueous dispersions of the nanoparticles with concentration *c* = 3.5 wt.%. Then, by successive dilutions, lower concentrations including *c* = 3.5, 2.5, 1.5, 0.75, 0.3 and 0.03 wt.% were obtained. All samples, at these concentrations and pH 6, showed a good colloidal stability over several weeks. All the hyperthermia measurements were performed under as similar as possible conditions, i.e. the sample holder (eppendorf vial) and its volume (300 µL), its position within the magnetic field, and measurement time (600 s). Three field frequencies (*f* = 137, 240, 339 kHz) with amplitude H_0 _= 7.5 kA/m were chosen in addition to a field dependence study at 339 kHz. Field parameters in all experiments were under the criteria of linear response theory and also satisfying the safety restriction introduced by Hergt *et al*.^48^.

A fiber optic thermometer system (Qualitrol NOMAD-Touch Portable Fiber Optic Monitor) was used to record the temperature of the samples with accuracy of ± 0.1 °C. To calculate the SLP values, the temperature versus time results were fitted using the Box–Lucas equation as:

7 $$T(t)=A(1-\exp (\,-\,Bt)),$$

where the product *AB* stands for the initial slop of the heating curves^53^. This is considered as one of the most accurate methods to calculate the SLP values for non-adiabatic experimental conditions^54^. Then the SLP values were calculated as:

8 $${\rm{SLP}}=\frac{{C}_{W}{m}_{W}+{C}_{np}{m}_{np}}{{m}_{np}}\frac{\Delta T}{\Delta t}{|}_{t\to 0},$$

where *m*_*W*_ and *m*_*np*_ are the masses and *C*_*W*_ and *C*_*np*_ are the specific heat capacity of water and nanoparticles, respectively. Although our experimental setup was non-adiabatic, by using sufficient insulators and water cooling of the coil, the temperature of the coil could be controlled with accuracy of  ± 0.5 °C; therefore, any evidence of peripheral heating caused by the coil was eliminated. This was tested using a blank sample (pure water in the same holder) and no temperature change was observed.

## Results

### Experimental results

The characteristics of the samples are listed in Table 1. The smaller saturation magnetization in sample x = 0 compared to bulk magnetite (446 kA/m)^36^ can be due to partial oxidation of the particles and size effects^32,55^. It has been shown in various studies^28,31,37,46,56^ that incorporation of zinc, up to certain amounts, in magnetite structure can considerably enhance the saturation magnetization of the composition. However, the peak for this enhancement, observed in x = 0.1 in our samples^32^, has been seen at different amount of zinc^28,31,37,46^. This is most probably due to different cation distribution in the composition which is very sensitive to parameters such as synthesis method and size of the particles^57,58^. The mean particle diameter (<*d* > ) from TEM images (results published in a previous study^32^) and the hydrodynamic sizes (*D*_h_) from DLS measurements show a relatively narrow size distribution. The sizes measured by DLS are larger than those measured by TEM. This difference in the mean sizes is expected due to both the hydration and the surface charge of the samples^59,60^. The zeta potential of the samples x = 0.0, 0.1, 0.2, 0.3, and 0.4 were 16.2 (5.9), 25.6 (3.4), 15.1 (5.9) 20.3 (5.1), and 24.7 (3.5) mV, respectively, where the numbers in parentheses are the zeta deviations. The zeta potential close to 20 mV indicates a relatively good colloidal stability for all samples, therefore, the larger size of the particles measured by DLS can be related to the hydration of the samples.

**Table 1:** Characteristics of the samples, the numbers in parentheses for < *d* > are the polydispersity degree calculated as (standard deviation/mean particle diameter) and for *D*_h_ are the polydispersity index (PDI) provided by the zeta-sizer instrument.

Sample	M_s_ (kA/m)^32^	<*d* > (nm)^32^	*D*_h_ (nm)	*K*_*eff*_ (kJ/m^3^)	*σ*	*τ*_*N*_ (s)	*τ*_*B*_ (s)
x = 0	270	13.7 (0.15)	85 (0.24)	23 ± 2	7.4	1.6E-06	2.3E-04
x = 0.1	425	12.4 (0.12)	75 (0.11)	27 ± 3	6.7	8.5E-07	1.6E-04
x = 0.2	291	9.6 (0.12)	75 (0.13)	43 ± 6	4.9	1.3E-07	1.61E-04
x = 0.3	255	6.5 (0.16)	40 (0.32)	56 ± 7	2	7.2E-09	2.4E-05
x = 0.4	251	5.3 (0.15)	55 (0.41)	65 ± 9	1.2	3.4E-09	6.3E-05

**Figure 1:**
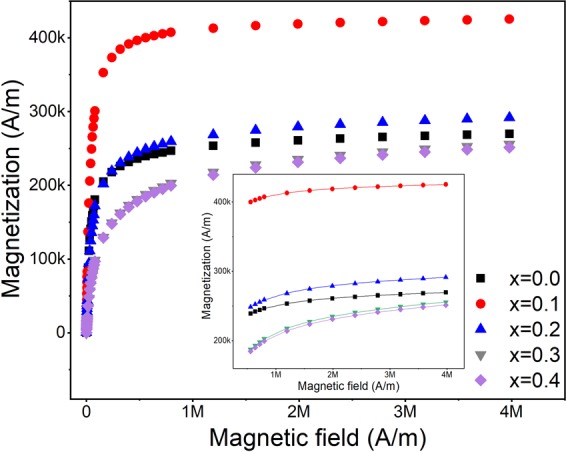
First quadrant of the magnetization curves of the samples. The inset is the fitted high-field magnetization data with the law of approach, the symbols are experimental data and the lines are the fittings.

The calculated effective anisotropy constants utilizing the law of approach to saturation and relaxation times of each sample are summarized in Table 1. Equation (6) fitted all experimental data with R-squared values higher than 98% (Fig. 1). Since the magnetization curves for samples x = 0.2, 0.3, and 0.4 were not completely saturated, *M*_*s*_ for these samples were estimated by plotting the high field values of *M* versus 1/*H* and the intercept of the line with the *M* axis (*H* = ∞) were chosen as *M*_*s*_. This behavior can be an indication for the presence of a higher anisotropy in these samples^61^. The obtained *K*_*eff*_ for sample x = 0 is slightly higher than that for bulk magnetite, 18 kJ/m^3^ ^62^, but very close to the other reports with the same particle size and prepared with coprecipitation method^63–65^. However, one should keep in mind that due to instability of the magnetite nanoparticles, especially prepared by coprecipitation method, there always exists a degree of oxidation in the surface of the particles, as it may form a core−shell composition of magnetite-maghemite^66,67^. Park *et al*.^55^ also observed a partial oxidation in magnetite nanoparticles of 12 nm prepared with thermal decomposition method and the estimated anisotropy were very close to ours. Recently, Modaresi *et al*.^68^ reported on the effect of zinc doping level on magnetic properties of ferrite nanoparticles (Zn_x_Fe_3−x_O_4_) prepared by coprecipitation method. In their study the effective anisotropy was also estimated using the law of approach and the result, contrary to ours, showed a direct relation between particle size and the effective anisotropy. However, all particle sizes in their study were considerably larger than the present study. For example, for sample x = 0.3 with particle size ~37 nm they estimated the effective anisotropy as 13.7 kJ/m^3^ (56 kJ/m^3^ in our results). Although the addition of zinc up to certain amounts in ferrites can result in a decrease in their effective anisotropy^69^, in our study, the effective anisotropy was increased by increasing the zinc content which is most probably due to the size effects^70^. Upon decreasing the nanoparticles size, here induced by increasing the zinc content, the surface effects become progressively dominant. Disorder in lattice symmetry close to and at the surface appears and disordered spins at the surface induce additional anisotropy^71,72^. It should also be mentioned that in an assembly of magnetic nanoparticles with certain size distribution, a distribution of anisotropy constants exists^64^. However, we considered the obtained values as the mean values for *K*_*eff*_ in all calculations.

**Figure 2:**
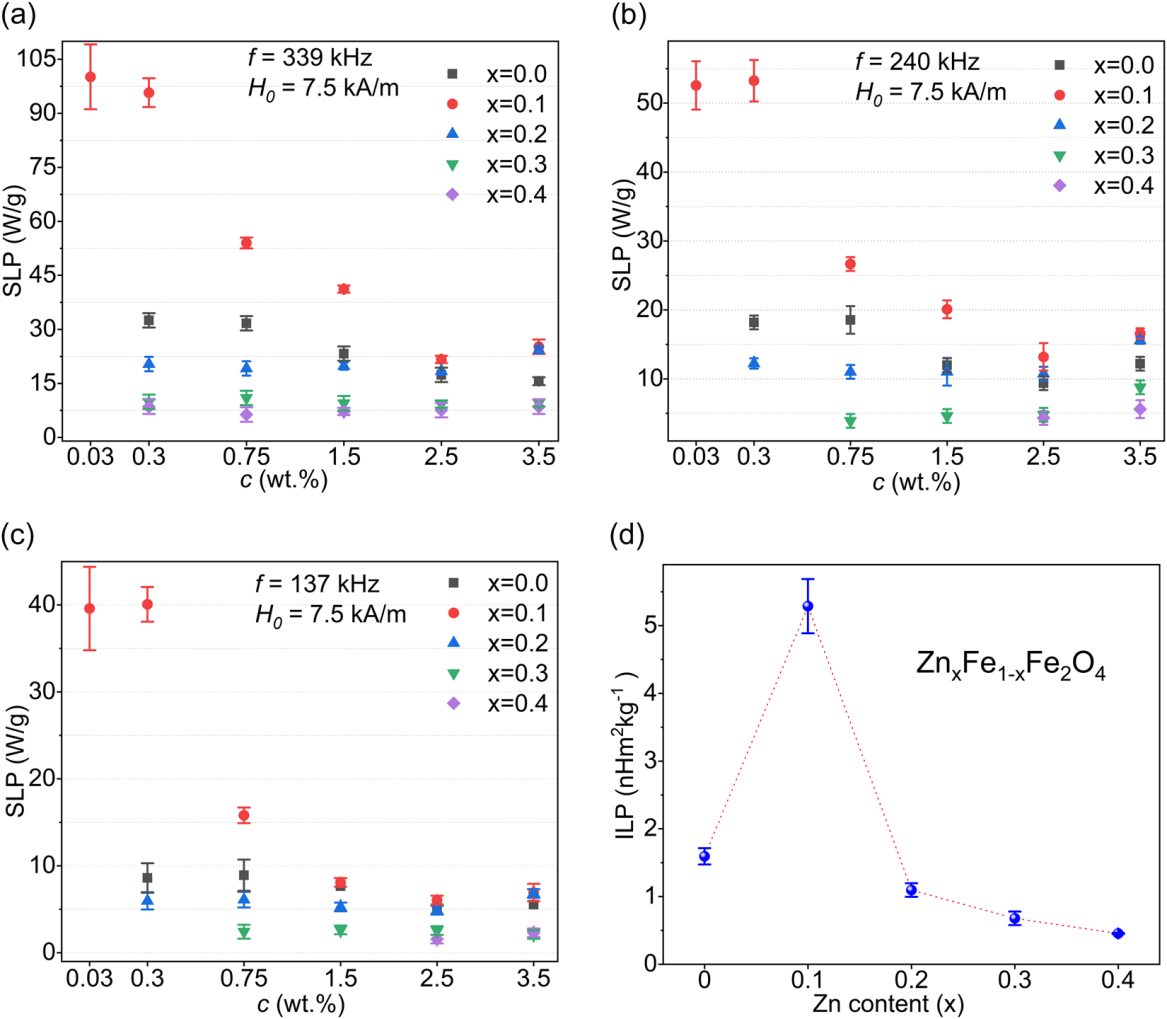
The measured SLP values of the samples at *H*_0_ = 7.5 kA/m and different concentrations for (**a**) 339 kHz, (**b**) 240 kHz, and (**c**) 137 kHz. (**d**) Mean ILP values considering all frequencies.

The heating efficiency of the samples was evaluated as a function of frequency and amplitude of the applied magnetic field as well as sample concentration. The latter determines interparticle distances in the colloidal system which in turn can affect the interparticle interactions. Figure 2 shows the concentration dependence of SLP at frequencies 137, 240, and 339 kHz. Although each sample at different frequencies showed similar trends by varying its concentration, we found significant different behaviors by changing the concentration of each sample for all frequencies. Samples x = 0.2, 0.3, and 0.4 showed almost no concentration dependence, while the SLP values for samples x = 0.0 was relatively dependent on concentration. The SLP values of this sample increased by a factor of ~1.5–2 from higher concentrations to the concentration *c* = 0.75% and then for lower concentrations no significant change was observed. The increase observed in the SLP of the sample x = 0.1 was dramatically more pronounced. It increased by a factor of ~5–6 from higher concentration to the concentration *c* = 0.3% and then it remained unchanged for lower concentrations. Meanwhile, it should be noted that only the sample x = 0.1 showed a measurable temperature change at very low concentration *c* = 0.03% and for sample x = 0.4, for instance, no considerable temperature rise was observed at concentrations lower than 2.5% for frequencies of 137 and 240 kHz.

When comparing the SLP values among the samples of each concentration for all frequencies, the sample x = 0.4, which has the smallest size and the highest anisotropy constant value, showed the lowest SLP values. The sample x = 0.1 has the highest saturation magnetization, but size and anisotropy constant close to that of sample x = 0. This sample was the most efficient sample for almost all concentrations and frequencies. The sample x = 0.2 with a higher saturation magnetization and anisotropy, but smaller size than x = 0, at higher concentrations showed a higher SLP value than that of x = 0. However, by decreasing the concentration, SLP of sample x = 0 overtook that of sample x = 0.2. These results show a very complex dependence of SLP on different parameters such as size, anisotropy, saturation magnetization, and concentration. Meanwhile, there are other important parameters such as size distribution^73^ or the effect of nanoparticle coating^74^ which has not been considered in our study.

The intrinsic loss power (ILP) was introduced by Kallumadil *et al*.^53^ to improve the comparison among the SLP values obtained using different magnetic field parameters. According to the linear response theory, ILP is calculated by normalizing the SLP values to *f* and $\,{H}_{0}^{2}$. ILP values for all samples are shown in Fig. 2 (d) with the highest value 5.4 nHm^2^kg^−1^ for the sample x = 0.1. Besides the exceptional high ILP value reported for natural bacterial magnetosomes (23.4 nHm^2^kg^−1^)^75^, for synthetic magnetic nanoparticles high values such as 8.7^76^, 7.4^77^, 6.1^78^, and 4.1 nHm^2^kg^−1^ ^15^ have been reported, all well above commercially available magnetic nanoparticles^53^. Values above 3 are considered as suitable for magnetic hyperthermia^79^.

**Figure 3:**
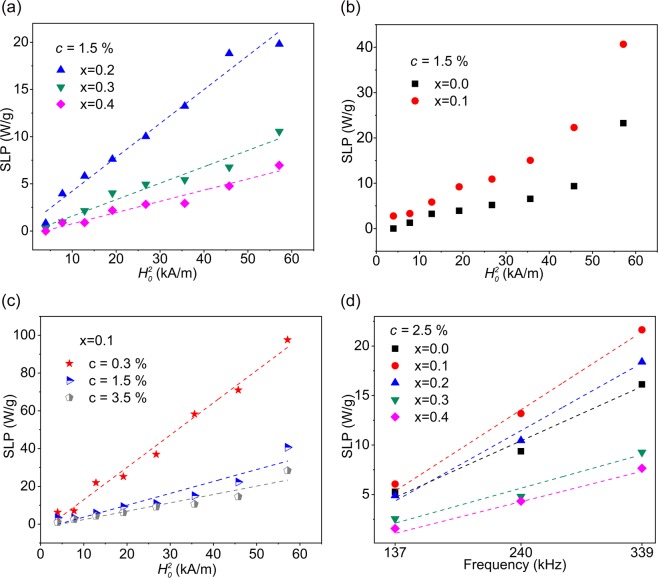
SLP field dependence (**a**) for sample x = 0.2, 0.3 and 0.4 and (**b**) for sample x = 0.0 and 0.1 for *c* = 1.5 wt.%. (**c**) SLP field dependence for sample x = 0.1 and *c* = 3.5, 1.5, and 0.3 wt.%. (**d**) SLP frequency dependence for all samples at *c* = 2.5 wt.%.

Since the criteria for linear response theory has been respected in all experiments of this study, SLPs are expected to be varied proportional to the square of the magnetic field amplitude. We chose the concentration *c* = 1.5% as a moderate concentration among our samples to study the field dependence of the SLP. The quadratic dependence was observed only for samples x = 0.2, 0.3, and 0.4 (Fig. 3(a)), but not for samples x = 0.0 and 0.1 (Fig. 3(b)). For this reason, sample x = 0.1 was examined for two more concentrations. As it can be seen in Fig. 3(c), only for the lowest concentration sample the quadratic field dependence was observed. Such discrepancy has been reported in other studies^80,81^ and will be discussed in details in the succeeding sections. However, the SLP variations for all samples and concentrations, as predicted by the linear response theory, were directly proportional to the frequency. Figure 3(d) shows an example of this frequency dependency for *c* = 2.5 wt.%.

### Numerical simulation results

**Figure 4:**
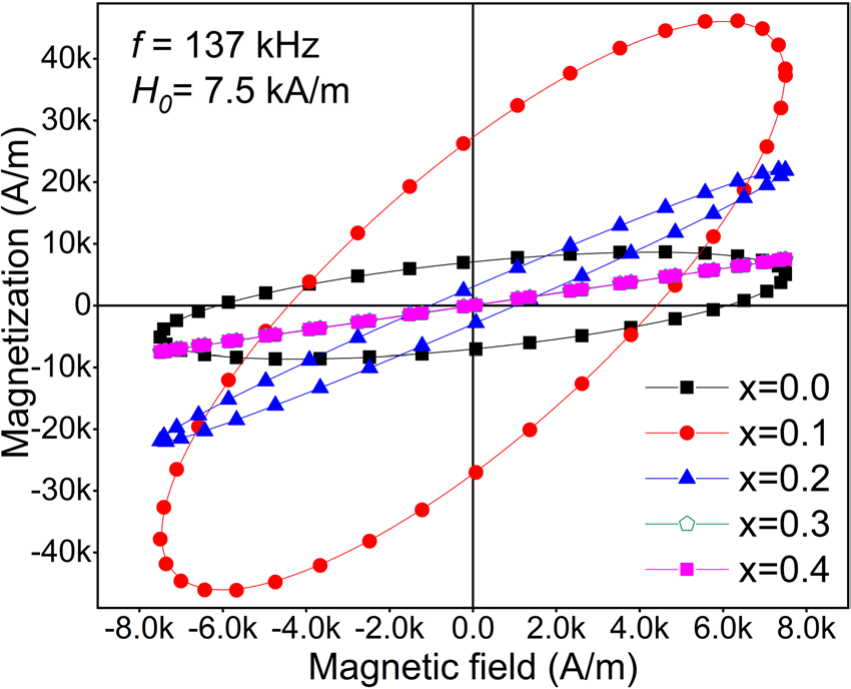
The dynamic hysteresis loops of the samples considering *f* = 137 kHz and *H*_0_ = 7.5 kA/m.

Figure 4 shows the dynamic hysteresis loops of the samples obtained using Eq. (5). Although all samples behaved as superparamagnetic in quasi-static fields^32^, at higher frequencies they show an open elliptical shape loop which is the result of the lag between the magnetization with respect to the applied alternating field. This lag for samples x = 0.3 and 0.4 is such small that results in extremely low SLP values.

**Figure 5:**
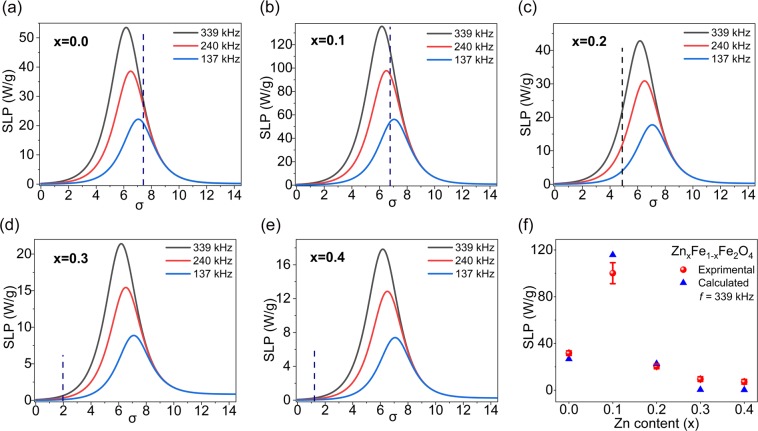
(**a**–**e**) SLP variation versus *σ* = *K*_*eff*_*V*/*k*_B_*T* considering the volumes obtained from TEM images (the dashed lines represent the experimental values), and (**f**) the calculated and experimental values of SLP for all samples for *f* = 339 kHz and at *c* = 0.3%.

The dimensionless parameter *σ* = *E*_*A*_/*k*_*B*_*T* = *K*_*eff*_*V*/*k*_*B*_*T* is an essential parameter directly influencing the relaxation time of the samples and hence their SLP values. Figure 5 shows the simulated variation of SLP versus *σ* for each sample at different frequencies by considering the volume of the nanoparticles obtained from TEM and a wide range of *K*_*eff*_ values. The dashed line in each figure represents the *σ* value of the corresponding sample using the estimated *K*_*eff*_ by the law of approach. Although the simulated SLP values were in good agreement with the experimental results at *c* = 0.3% (see Fig. 5(f) as an example for *f* = 339 kHz), one should keep this in mind that these SLPs have been calculated considering the inverse attempt frequency of 10^−9^ s for all samples which is only a rough approximation of this parameter. Because *τ*_0_ strongly depends on intrinsic properties of the magnetic nanoparticles^14^ and for each sample should be different. Moreover, the calculated results for samples x = 0.0 and 0.1 had more discrepancy at higher concentration due to increasing the dipolar interactions.

**Figure 6:**
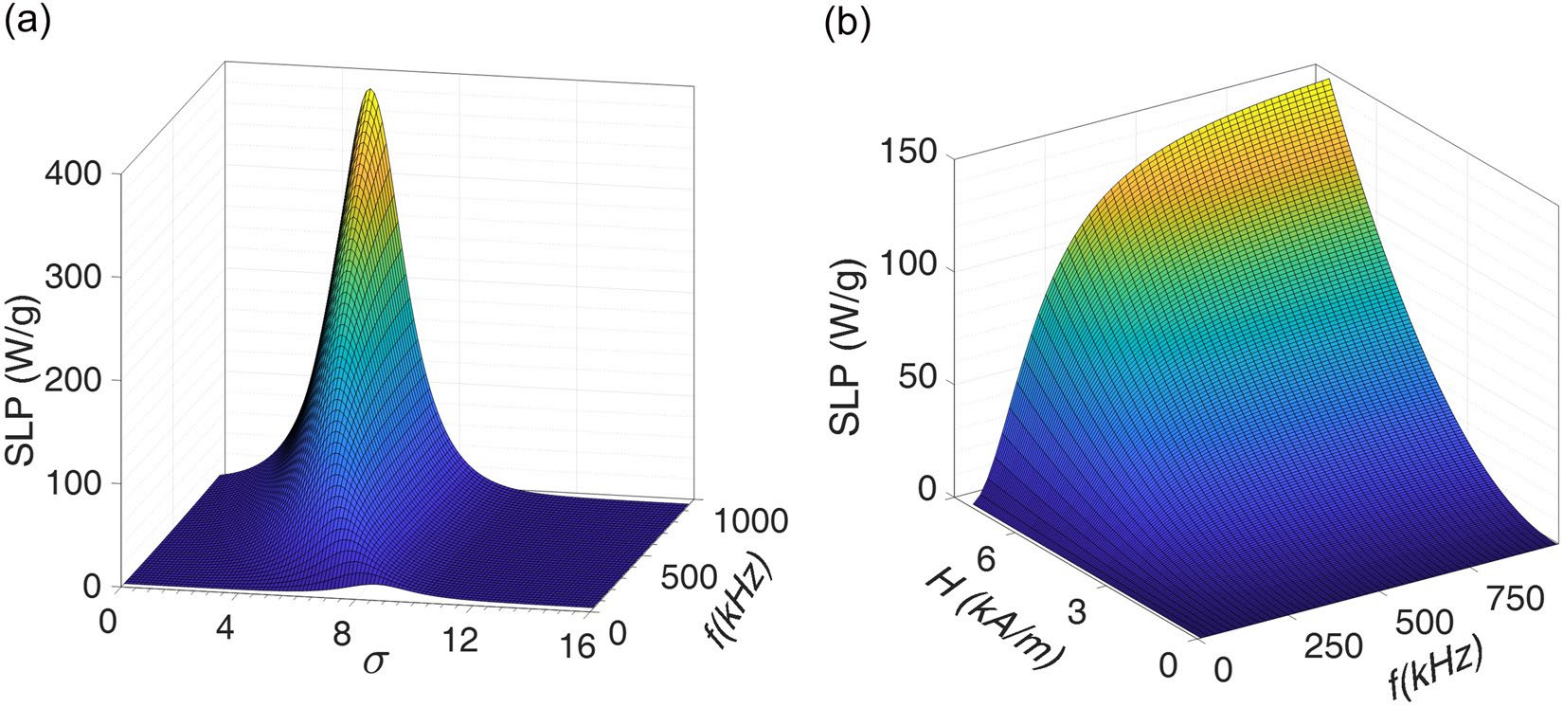
Simulated SLP versus (**a**) *σ* and frequency for *H*_0_ = 7.5 kA/m, and (**b**) field amplitude and frequency for *σ* = 6.7.

As it can be clearly seen in Fig. 5, there is a maximum SLP for each frequency which is shifted to lower values of *σ* at higher frequencies. This is better shown in Fig. 6 where the SLP dependence on *σ* and frequencies up to 1 MHz is plotted for sample x = 0.1. SLP increases by increasing the frequency and its maximum occurs at different *σ* for each frequency. However, at a fixed value of *σ*, SLP saturates at higher frequencies and it can be immediately understood from Eq. (2) for 2π*fτ* ≫ 1^82^. This can be visualized in Fig. 6(b) as an example that shows the SLP dependence on frequency and field amplitude for the sample x = 0.1 with *σ* = 6.7.

## Discussion

The linear response theory was chosen for numerical simulations since all experiments in this study were conducted at relatively low field amplitudes, which respect the condition *µ*_0_*H*_0_*M*_*s*_*V* ≤ *k*_*B*_*T* for all samples. Moreover, as it was pointed out by Ruta *et al*.^83^, the ratio between *H*_0_ and the anisotropy field (*H*_*K*_ = 2 *K/µ*_0_*M*_*s*_) of the sample can also determine the applicability of the linear response theory. For values *H*_0_/*H*_*K*_ ≪ 1, the linear response theory can quantify the experimental results. In the present study, for all samples this ratio was in the order of 10^−2^.

### Interparticle interactions

In the framework of the linear response theory, in addition to assuming a monodispersed magnetic nanoparticles system, no contribution from dipolar interactions between the particles has been taken into account. However, in real conditions these two assumptions are rarely met and there may always exist a degree of polydispersity^84^ and also interparticle interactions. Here, we assume the first assumption is true and try to interpret the observed results with the hypothesis that the variations in the SLP values with concentration are related to interparticle interactions. This hypothesis is relevant, especially because interparticle interaction strongly depends on interparticle distances and this varies with the concentration in a colloidal dispersion.

As mentioned above, apparently very different theoretical and experimental results regarding the effect of interparticle interactions have been reported and this discussion still appears to be not conclusive. For example, in two very recent studies interparticle interactions have shown to significantly increase^85^ and decrease^86^ the heating efficiency of the system. In our results, however, two distinguishable trends can be recognized. For sample x = 0.0 and 0.1, decreasing the concentration, which means increasing the interparticle distances and hence reducing the strength of the interactions, has led to an increase in their SLP values. On the other hand, for the other three samples their heating efficiencies have not been influenced by this effect. Presa *et al*.^20^, using maghemite nanoparticles with different sizes, showed that even from extremely high concentrated sample (~30%) to the low concentration of 0.6%, the SLP values remained unchanged. They supposed that either the dipolar interactions are not significant to influence the heating efficiency or due to the existence of clusters, dilutions will not change the effect of the interactions. We believe in our results the former explains the monotonous behavior observed in samples x = 0.2, 0.3, and 0.4. This will be discussed in more details in the following paragraphs.

Although most of the theoretical studies show a negative effect of interparticle interaction on hyperthermia performance of magnetic nanoparticles^14,17,18,87^, several studies have shown that depending on intrinsic properties of the nanoparticles, interactions may increase or decrease the heating efficiency of the system^9,80,88^. Tan *et al*.^9^, for example, using Monte Carlo simulations showed that the heating efficiency of the nanoparticles, depending on the relation of the applied magnetic field with the coercivity or saturation field of the nanoparticles, may increase, decrease, or behave in a way they called as bell shape. They also showed the effect of interactions is more pronounced for magnetic nanoparticles with lower anisotropy. In references^10,19,22^, the authors have shown that for certain sizes and shapes of the formed clusters or chains in colloidal dispersions, SLP may increase or decrease. They showed that for clusters that are anisotropic in shape, there will be an extra anisotropy induced to the system leading to an increase in the SLP values. While an isotropic configuration has a negative influence on the heating efficiency compared to non-coupled nanoparticles. For samples x = 0.0 and x = 0.1 of the present study, we also believe the pronounced effect of interparticle interactions arising from the large magnetic moments of their single domain particles can explain the observed behavior.

**Figure 7:**
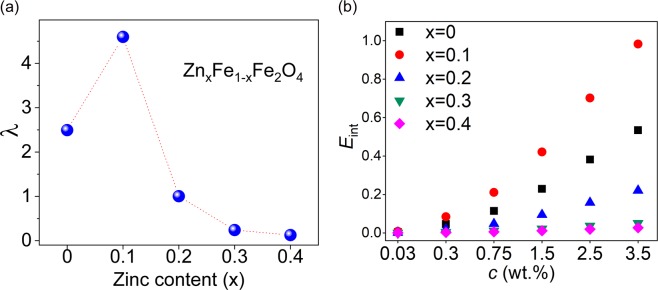
(**a**) Dipolar coupling constant (λ) and (**b**) relative strength of the dipolar interaction at different concentrations.

In a colloidal dispersion of single domain magnetic nanoparticles with the magnetic moment *µ* = *VM*_*s*_, the maximum magnetic interaction potential between two neighboring particles in zero applied magnetic field is given by^84^:

9 $${U}_{\max }=\frac{{\mu }_{0}{\mu }_{i}{\mu }_{j}}{2\pi {d}_{ij}^{3}},$$

where *d*_*ij*_ = (*d*_*i*_ + *d*_*j*_)/2 with *d*_*i*_ and *d*_*j*_ the diameters and *µ*_*i*_ and *µ*_*j*_ are the magnetic moments of the particles *i* and *j*. To characterize the interparticle interactions, the parameter *λ* has been introduced as dipolar coupling constant which relates *U*_max_ for two particles in contact with one another to the thermal energy as:

10 $$\lambda =\frac{{\mu }_{0}{\mu }^{2}}{2\pi {d}^{3}{k}_{B}T},$$

where *µ* and *d* are the mean magnetic moment and mean diameter, respectively. For *λ* > 2 the effect of the interactions is non-trivial, the system is considered as strong interacting, and aggregates may happen. When *λ* < 2 the system is said to be in homogenous “gaslike” state and the interparticle interactions are negligible^84^. The calculated λ values for our samples (Fig. 7(a)) show that only samples x = 0.0 and 0.1 are within the strong interacting limit.

Moreover, a qualitative analysis of the interparticle interaction energy at different concentrations can also demonstrate how pronounced the effect of interaction for the samples x = 0.0 and x = 0.1 is compared to the other three samples. The maximum magnitude of the dipolar interaction energy (normalized to the thermal energy) of two neighboring particles with the mean interparticle distance *r* from one another can be expressed as^89^:

11 $${E}_{\mathrm{int}}=\frac{{\mu }_{0}{\mu }^{2}}{4\pi {r}^{3}{k}_{B}T}.$$

By assuming perfect and rigid spherical particles, the interparticle distance of a colloidal dispersion sample with volume *V*_*s*_ and consisting of *N* particles can be obtained as *r* = (*V*_*s*_/*N*)^1/3^. Although Eq. (11) considers only two neighboring particles, it can qualitatively be an indication of the magnitude of the interparticle interactions in the whole system. Quantitative comparison implies more considerations and extensive computational methods (see for example^17^) which is beyond the scope of the present study. Considering all concentrations and using the mean particle diameters of our samples in the volume of 300 µL, *E*_int_ was calculated. Figure 7(b) shows this quantity (relative strength of the dipolar interaction) for all samples at all concentrations. As it can be seen, interactions in samples x = 0.0 and 0.1 are higher than the other three samples at higher concentrations and at the lowest concentration all samples seem to act as non-interacting particles.

According to the preceding discussions, we can conclude that two different trends of heating efficiencies versus concentration can be related to the different effect of interparticle interaction in the samples. As stated earlier, it has been proven that chain and cluster formation in interacting particles will introduce an additional anisotropy to the system^14,19,22,23^. Moreover, theoretical and experimental evidences have shown that interparticle interaction considerably increases the energy barrier in single domain magnetic nanoparticles^49,90–92^. Having this in mind and recalling the results shown in Fig. 5, the reason for the observed trends in SLP values can be qualitatively understood by considering: i) the calculated SLP values versus the parameter *σ* has been obtained using the linear response theory without considering the effect of interparticle interactions and ii) the increase in the energy barrier directly leads to a shift in parameter *σ* to higher values. This effect can be qualitatively evaluated for sample x = 0.0 and 0.1 in Fig. 5 by shifting the dashed lines to the right side for higher concentrations. In fact, here in samples x = 0.0 and 0.1 increasing the parameter *σ*, increases their relaxation times and hence leading to more deviation from the maximum heating efficiency condition of 2*πfτ* = *ωτ* = 1 at each given frequency^93^. For these two samples, the product *ωτ* is higher than 1 for all frequencies except for *f* = 137 kHz sample x = 0.1; though close to 1. These results are in complete agreement with previous theoretical study by Landi based on a mean-field model^8^.

Although for the other samples increasing the parameter *σ*, apparently, should have a positive effect on their SLP, such an effect was not clearly observed. This is most likely due to the negligible strength of the interparticle interactions in these samples. Tan *et al*.^9^, in their theoretical study on the effect of interparticle interactions, related the SLP values to the dimensionless concentration parameter expressed as ${\mu }_{0}{M}_{s}^{2}c/{K}_{eff}$. In their results, for values higher than 0.02, interparticle interactions became noticeable. In our experimental results, SLP for sample x = 0.0 at concentrations below *c* = 1.5% and sample x = 0.1 at concentrations below *c* = 0.75% was almost not affected by concentration. The corresponding calculated dimensionless concentration for these two conditions is 0.06 which can be considered as the threshold value for our samples. Dimensionless concentration for all other samples, at all concentrations, were well below 0.06 except for sample x = 0.2 at *c* = 3.5% which is around 0.06.

Another feature of the experimental results, which may be explained by considering the interparticle interactions, is the discrepancy from quadratic field dependence of SLP values at higher concentrations for samples x = 0.0 and 0.1. Carrião *et al*.^94^ showed that depending on the changes in the parameter *σ* and hence the relaxation time from its optimum value (*ωτ* = 1), different deviation from the quadratic field dependence may happen. Here, a qualitative analysis can also be conducted using the observed field dependence of SLP for sample x = 0.1 at two concentrations *c* = 3.5% and 1.5% in order to show the changes in *σ*. By simulating the SLP(*H*_0_) variation versus *σ* using the same field amplitudes as in the experiments and then correlating these simulations with the observed experimental SLP(*H*_0_) values, new values for parameter *σ* can be estimated. The estimated mean values were 8.4 ± 0.2 and 8.9 ± 0.2 for concentrations *c* = 1.5% and 3.5%, respectively, which shows a considerable increase due to the interparticle interactions compared to that obtained using *K*_*eff*_ of this sample (*σ* = 6.7). However, it is worth noting that from these values no clear conclusion about the induced anisotropy to the system can be drawn, because at the presence of the applied magnetic field, the height of the energy barrier is the result of the relative interplay between the anisotropy, Zeeman, and interaction energy in a complex manner^91^. Therefore, when employing the linear response theory to interpret the experimental results, in addition to respecting the defined limit for linear regime i.e. *µ*_0_*H*_0_*M*_*s*_*V* ≤ *k*_*B*_*T*, a good knowledge of interparticle interactions seems to be crucial too.

### Heating efficiency

Overall, the samples under study here present a range of different intrinsic properties. The results show that their heating efficiency does not depend on a single intrinsic property, but on a variety of parameters. Interpreting the results considering only one parameter, for example, size or anisotropy may be misleading. These two characteristics, especially at nanoscale, do not vary independently of one another. Instead, the parameter *σ*, which is a deterministic quantity for determining the relaxation time of the particles, is more appropriate to be discussed. In a fixed field frequency and amplitude, for a magnetic nanoparticles system with a known saturation magnetization, and in linear regime this parameter determines the heating efficiency of the system; i.e. how close the system is to the condition *ωτ* = 1. In our samples, for example, although there is no considerable difference in the saturation magnetization of samples x = 0.0 and x = 0.3 and 0.4, their SLP values are considerably different. This can be seen from the position of their *σ* in the graphs shown in Fig. 5. On the other hand, samples x = 0.0 and x = 0.1 present similar *σ* values, and the dramatic difference in their heating efficiency arises from the difference between their saturation magnetization values. The importance of the parameter *σ* can be better understood by comparing the SLP values of sample x = 0.0 and 0.2, where the latter has a higher saturation magnetization but a smaller *σ*. The sample x = 0.0, in most of the concentrations studied, showed a better performance than x = 0.2.

**Figure 8:**
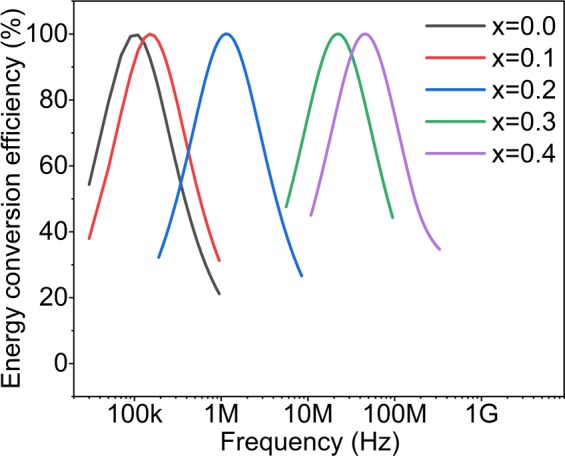
Energy conversion efficiency versus frequency for all samples.

As a final remark, we recall the results shown in Fig. 6(a) as a typical example of the dependence of SLP on the field frequency and *σ*. By increasing the field frequency, the peak positions in this figure are shifting to the lower *σ* values. Considering the parameter *σ* as a fixed intrinsic constant, the condition *ωτ* = 1 occurs only at a certain frequency for each kind of magnetic nanoparticles. Therefore, any frequency higher or lower than this frequency leads to a less efficient energy conversion. Figure 8 shows this energy conversion efficiency for all samples analyzed in this study. As it can clearly be seen, only for samples x = 0.0 (at 100 kHz) and 0.1 (at 150 kHz) the maximum energy conversion efficiency can occur at usable frequencies for clinical trials. Moreover, the larger effective anisotropies in samples x = 0.2, 0.3, and 0.4 imply higher field amplitudes to achieve a reasonable SLP value and this is also another limitation for these kind of samples to be used in clinical magnetic hyperthermia. In such situations, due to safety considerations, increasing the field frequency or amplitude is not possible; therefore, tuning the intrinsic magnetic properties of the nanoparticles is the preferred strategy.

Cation substitution in ferrite nanoparticles has always been considered as a very straightforward strategy to optimize their structural and magnetic properties. In the present study, zinc-substituted magnetite (sample x = 0.1) showed superior SLP values compared to the magnetite nanoparticles with a relatively high ILP value. In our previous study^32^, this composition improved the signal to noise ratio in magnetomotive ultrasound imaging technique as a diagnostic application, which shows the great potential of the sample x = 0.1 to be considered as a theranostic agent in biomedical applications. Indeed, theranostic is the fusion of diagnostic and therapeutic procedures in a single nanoparticles system where the same magnetic nanoparticles can serve, simultaneously, as an image contrast agent and to deliver targeted chemotherapeutic agents directly to the cancer cells or as heating mediator in magnetic hyperthermia^95,96^.

## Conclusion

In summary, zinc-substituted magnetite nanoparticles were examined in various experimental conditions for magnetic hyperthermia. Due to different intrinsic properties of these compositions, they showed to be a very useful tool to observe the effect of parameters such as saturation magnetization, anisotropy and size on the heating efficiency of the magnetic nanoparticles. The sample Zn_0.1_Fe_0.9_Fe_2_O_4_ showed a relatively high ILP value (5.4 nHm^2^kg^−1^) compared to the other reports on Zn_x_Fe_1−x_Fe_2_O_4_ composition in the literature (0.5^46^, 1.57^97^, and 2.36^31^ nHm^2^kg^−1^), which offers a great potential to be considered as a theranostic agent. Although Srivastava *et al*., recently, reported ILP values ranging from 1.16 to 13.76 nHm^2^kg^−1^, comparing their results with ours is quite tricky. Because ILP parameter is only valid in the framework of linear regime, while in that study high fields up to 20 kA/m were used. Furthermore, the different intrinsic properties had diverse effects related to the dipolar interactions on the heating efficiency of the particles. For linear regime, this study resulted in two main conclusions: i) at the same sample concentration the strength of dipolar interactions is determined by the intrinsic properties; ii) if the dipolar interactions are strong enough to affect the heating efficiency, the parameter *σ* determines the type of the effect. As interactions increase the parameter *σ*, for any *σ* value resulting in *ωτ* < 1, interactions can increase the heating efficiency. On the other hand, for *ωτ* ≥1 interactions have the opposite effect. This shows also the importance of choosing the appropriate frequency to increase the heating efficiency of high interacting magnetic nanoparticle systems. We believe these results, considering the wide range of intrinsic properties and experimental conditions investigated, may have important implications for optimizing the heating efficiency of magnetic nanoparticles in hyperthermia.

## Data Availability

The datasets generated and analysed during the current study are available from the corresponding authors on reasonable request.
